# Robotic Upper Limb Rehabilitation after Acute Stroke by NeReBot: Evaluation of Treatment Costs

**DOI:** 10.1155/2014/265634

**Published:** 2014-04-23

**Authors:** Masiero Stefano, Poli Patrizia, Armani Mario, Gregorio Ferlini, Roberto Rizzello, Giulio Rosati

**Affiliations:** ^1^Rehabilitation Unit, Department of Neurosciences, University of Padova, via Giustiniani 1, 35128 Padova, Italy; ^2^Medical Management, Eremo Hospital, Arco (Trento), Italy; ^3^Neurorehabilitation Unit, Eremo Hospital, Arco (Trento), Italy; ^4^Clinical Epidemiology Unit, Provincial Agency for Health Services of Trento, Italy; ^5^Department of Management and Engineering, University of Padova, Italy

## Abstract

Stroke is the first cause of disability. Several robotic devices have been developed for stroke rehabilitation. Robot therapy by NeReBot is demonstrated to be an effective tool for the treatment of poststroke paretic upper limbs, able to improve the activities of daily living of stroke survivors when used both as additional treatment and in partial substitution of conventional rehabilitation therapy in the acute and subacute phases poststroke. This study presents the evaluation of the costs related to delivering such therapy, in comparison with conventional rehabilitation treatment. By comparing several NeReBot treatment protocols, made of different combinations of robotic and nonrobotic exercises, we show that robotic technology can be a valuable and economically sustainable aid in the management of poststroke patient rehabilitation.

## 1. Introduction

Stroke has a high social impact because it is a leading cause of motor impairment and disability in ADLs [1]. In 85% of stroke survivors the recovery is partial [2], while in 35% of them a serious disability remains. The 30–60% of patients treated with traditional rehabilitation has a residual functional impairment of the paretic arm and consequently a reduction of ADLs is common [3, 4]. The ageing of population implies that an increasing number of people require rehabilitation after stroke [5, 6]. In the acute and subacute poststroke phases, the robot-assisted rehabilitation of the upper limb may be successfully used in alternative to conventional mobilization: in fact it results as effective as the conventional therapy, especially when it is used in addition to nonrobotic techniques [7–9]. In 2010, the average expense per person for stroke care was estimated at $5455 in the USA while the mean lifetime cost of ischemic stroke was estimated at $140 048 (including inpatient care, rehabilitation, and follow-up care necessary for lasting deficits) [1]. During 2001 to 2005, the average cost for outpatient stroke rehabilitation services and medications the first year after inpatient rehabilitation discharge was $11 145. The corresponding average yearly cost of medication was $3376, whereas the average cost of yearly rehabilitation service utilization was $7318 [1].

Despite the great diffusion of studies on the robotic therapy [10], few data are available about the real costs of robot-aided rehabilitation. Wagner et al. [11] analysed the usual care cost for stroke patients in comparison with additional intensive rehabilitation or additional robotic intervention. They stated that patients in the robot and intensive comparison groups had lower average costs than patients in usual care group, but there were not differences between robotic and intensive groups.

With the present study, we aim to complete our previous studies conducted on the use of the Neuro-Rehabilitation-Robot (NeReBot) for the treatment of poststroke upper limb impairment [7–9, 12], by presenting an evaluation of the costs related to delivering such therapy to patients, with reference to the standard costs of stroke rehabilitation in the Italian National Health Care System. Further work will be needed to obtain a complete cost-effectiveness evaluation of NeReBot treatment, though, this study allows comparing the costs of such therapy to the control therapies used in the NeReBot clinical trials run so far.

## 2. Methods

### 2.1. Description of Robotic Device

Our previous studies were conducted on the clinical utilization of the NeReBot, a device for the treatment of poststroke upper-limb impairment designed and developed at Padua University [13, 14]. This robotic device, unlike the other rehabilitation robots described in the literature, is based on direct-drive wire actuation. This solution can provide many benefits compared with devices characterized by a rigid structure, that is, lower costs, reduced complexity (spatial movements can be obtained despite the limited number of degrees of freedom), compliance by design [15], and a higher degree of reliability and safety. The device can (a) perform spatial movements (flexion and extension, pronation and supination, adduction and abduction, and a circumduction-like movement) of shoulder and elbow, (b) be easily moved to the hospital room and used for early training of the upper limb after stroke, and (c) be used to intervene on patients not only in the sitting but also in the supine position [8].

### 2.2. Participants

NeReBot was tested in two different clinical trials [8, 9, 12]. Both studies involved hemiparetic subjects in the acute and subacute phases of their stroke, enrolled within 15 days after stroke.

### 2.3. Study Design

The two randomized controlled trials tested two different robotic protocols in comparison to standard rehabilitation treatment, one using the robot in addition to the traditional treatment [8], one in partial substitution to the standard rehabilitation programme, with a dose-matched approach [9, 12]. Both protocols lasted 5 weeks and included two daily sessions of robotic treatment for five days a week.

### 2.4. Evaluation of Participants

Muscle tone (Modified Ashworth Scale), strength (Medical Research Council), synergism (Fugl Meyer motor scores), dexterity (Box and Block test and Frenchay Arm test), and ADLs (Functional independence Measure) were measured at all the evaluations (before, at the end of the treatment and at the follow up). As reported in [12], no significant between-group differences were found with respect to demographic characteristics, motor, dexterity, and ADLs, so the substitutive treatment protocol with NeReBot could be considered comparable to the traditional one. On the other hand, the additional protocol yielded greater gains in the robotic group, with respect to controls, both on functional and on motor scales [8]. In other words, patients treated with the Additional Protocol developed a greater recovery of motor function and coordination than patients treated only with the conventional protocol, both at the end of therapy and at follow-up (8 months).

### 2.5. Data Considered for Cost Evaluation

To evaluate the costs of robotic and control treatment in the acute and subacute phases poststroke, we considered the hourly cost of a therapist and the daily cost of hospitalization in a Rehabilitation Unit (both referred to the Italian National Healthcare System) and the hourly cost of the NeReBot (Table 1). The latter was derived considering the total purchase cost of the equipment (€50,000) and maintenance costs (8% of the purchase price, from the second year). Such costs were divided by the hours of operation, considering a five-year amortization period and a usage of 2080 hours per year. We assumed a use of the robot within 5 days of the week, 52 weeks per year, and 8 hours per day, for a total of 260 days of use per year (these data refer to the normal organization of the work of a rehabilitation service).

### 2.6. Intervention Costs (Therapist)

The hourly cost of the physiotherapist is obtained by dividing the annual cost of the physiotherapist (gross of social security and tax) for a number of hours actually worked at the rate of 8 hours per day, for 5 days a week, for 44 weeks per year (total: 220 working days).

### 2.7. Cost for Robotic Session

The hourly cost of a robotic session is given by the sum of two terms: the cost of using the equipment and the cost of the operator (physiotherapist), who makes setting up and supervision during robotic treatment. The last cost depends on the degree of supervision required, which depends on the degree of patient autonomy (greater supervision needed in the early stages of rehabilitation, gradually reduced in the following weeks).

In the case of the NeReBot, based on our clinical experience, we hypothesize that in the first week of treatment a reasonable level of supervision is 1 : 1 (one therapist, one robot), in the second week a level of 1 : 2 (one therapist, two robots), in next three weeks a 1 : 3 level, and a 1 : 4 level from then on. Thanks to the ease of use, the NeReBot requires few minutes to be set up at the beginning of the session (less than 5 minutes) and less time to change the exercise type. Therefore, a single physiotherapist can manage up to four robots, provided these are conveniently located in the same room.

### 2.8. Cost for Global Therapy

The cost of each protocol is obtained taking the sum of all conventional and robotic treatment sessions. The latter are evaluated on a weekly basis, by multiplying the weekly hours by the hourly cost of robotic therapy for the week in question, in the event of different levels of supervision by the therapist among weeks, as previously assumed.

## 3. Results

Based on a cost of about €33,000 gross per year, we get an hourly cost of therapist close to €19 (Table 2). Considering 30-minute robotic treatment sessions (to be repeated twice a day), according to the four different levels of supervision (from 1 : 1 to 1 : 4, i.e., 30, 15, 10, and 7.5 minutes per session), we get an hourly treatment cost of robotic therapy ranging between €25 (first week) and €11 (last weeks), according to the different impact of the cost of the operator with respect to the hourly cost of the equipment (Table 3).

### 3.1. Additional Protocol

The additional Protocol adopted in the first clinical trial [8] consisted of two daily robot-assisted rehabilitation sessions of 25 minutes each, 5 days per week for 5 weeks. The overall treatment consists of approximately 21 hours of robot-assisted exercises, starting during the first week after stroke, in addition to conventional rehabilitation. The additional hours are concentrated in the initial phase (first 5 weeks) of the conventional protocol, which has a total length of 8 weeks on average.

As shown in Table 4, the weekly cost of the additional robotic treatment is obtained by multiplying the weekly additional hours (4,17 hours/week) by the hourly cost of robotic therapy for the week in question, which varies according to the different levels of therapist supervision as previously assumed. By taking the sum of the weekly costs for the whole protocol, we get a total cost per patient just under €330.

Given the greater patient recovery reported in the clinical trial [8], one could expect to make an early dehospitalization of robotic patients, compared to the 8 weeks expected on average. Considering the daily hospitalization cost of €273.64 provided by Diagnosis Related Groups (DRG) in the Autonomous Province of Trento, comparable to the mean Italian DRG value, the recovery of the additional treatment costs could be achieved with an average degree of dehospitalization of 1.2 days per patient (e.g., by reducing the hospitalization of all patients by 1 day and by discharging a patient every five discharged 2 days earlier than the average). Any further reduction of patients' hospitalization would result in money savings for the Healthcare System.

### 3.2. Mixed Protocol

Another approach could be that of designing a mixed protocol, including some additional robotic sessions with respect to standard rehabilitation treatment and some substitutive robotic sessions in which part of the standard treatment is replaced by robotic treatment. This choice is supported by the result that robotic therapy with NeReBot can be effectively used also in partial substitution of the traditional treatment [9, 12].

Since in the last weeks of treatment the robotic treatment costs are lower than standard treatment, the cost of a mixed protocol may benefit from including additional robotic sessions in the last weeks, whose savings may compensate for the additional costs, with respect to standard treatment, related to the additional robotic sessions. On the other hand, the introduction of additional robotic sessions should lead to faster/greater recovery of patients [8]. As an example, we consider the possibility of modifying the traditional rehabilitative treatment as follows.

1. During the first period, which represents the phase of the greatest recovery, the protocol could be additional, in order to intensify treatment; this would imply additional costs if compared to conventional treatment alone.
2. In a second phase, the robotic treatment could be administered in substitution of the portion of conventional treatment dedicated to the upper limb. In this phase, the lower cost of robotic treatment could allow recovering the additional costs generated in the first phase.

Table 5 shows a hypothesis of 2 weeks of addition, with two daily robotic sessions of 25 minutes each, for 5 days per week (125 minutes), yielding a total of more than 8 hours of additional robotic treatment, with a cost per patient of about 170€. This cost is recovered with less than six weeks of substitutive robotic treatment, thanks to a differential (traditional-robotic) hourly cost of approximately €7 on average (Table 6).

## 4. Discussion

In this paper we provided a quantification of the costs of the additional treatment protocol tested in our first randomized controlled trial of NeReBot [8]. We also calculated the costs of a mixed protocol, including both additional and substitutive robotic training, which has never been tested on patients. In the proposed mixed protocol, without any additional cost with respect to conventional rehabilitation, patients would receive, in the subacute phase, more than 20% additional treatment time (8 hours) with respect to the one normally delivered to the proximal upper limb (40 hours within 8 weeks). The intensification of treatment in the first weeks is expected to bring greater gains on both functional and motor scales, on the basis of the results of the first clinical study [8]. On the other hand, the partial replacement of conventional treatment with robotic therapy is not going to lead to alterations in motor and functional recovery [9, 12]. Therefore, not only with the Additional Protocol but also with the Mixed Protocol we can expect to be able to anticipate the discharge of patients, which will generate savings for the National Health Care System.

Robotic technology can be a valuable aid in the management of poststroke patient rehabilitation. From the study carried out, we can conclude that the costs of such interventions can be considered easily affordable, if delivered through easy-to-use and moderate-to-low cost devices. Potentially, by implementing adequate rehabilitation protocols, those costs may even be eliminated, while bringing significant economic benefits as well as better clinical outcomes at the same time.

Considering the huge social impact of stroke [1] and the aforementioned benefits and sustainable costs of a robotic treatment, we think that more efforts should be spent to invest in robot-assisted treatment and to study and test novel treatment protocols, also from a cost-effectiveness point of view, in order to well direct the economic resources improving the rehabilitation treatment. The number of people who could take benefit from robotic treatment is expected to grow in the coming years [5, 6], and hospitals that use robotic technology for stroke rehabilitation certainly will have more instruments to answer the growing rehabilitation needs.

Certainly, our model has some limitations, and to be reproduced in other settings requires the availability of more than one robot, adequate working space, and trained physiotherapists. Moreover, a thorough evaluation of cost-effectiveness of our treatment approach needs further work, as we provided so far only clinical evidence and evaluation of treatment costs, which are only part of the story. More in general, some questions still remain open in this field and more studies will be needed in the near future. Hypothetically, reaching a better functional recovery and reduced residual disability should bring also a reduction of the lifetime cost of stroke, but more research is needed to state it. Further scientific effort should be spent to evaluate if the robotic treatment can be economically advantageous not only in the acute and subacute phases but also in the chronic phase.

## Figures and Tables

**Table 1:** Hourly robot cost.

Hourly robot cost	
Robot purchase value	€50.000,00
Annual maintenance (from the 2nd year)	€4.000,00
Amortization period (years)	5
Total robot cost	€66.000,00
Annual robot cost	€13.200,00
Effective days of use per year	260
Daily working hours	8
Annual working hours	2.080
*Hourly robot cost *	*€6,346 *

**Table 2:** Hourly physiotherapist cost.

Hourly physiotherapist cost	
Gross yearly cost	€33.040,00
Effective working days per year	220
Daily working hours	8
Annual working hours	1.760
*Hourly physiotherapist cost *	*€18,773 *

**Table 3:** Hourly robot-aided therapy cost (including physiotherapist cost).

Hourly robot-aided therapy cost (including physiotherapist cost)			
Number of robots per therapist	Length of session (min)	Therapist supervision (min)	Hourly cost (robot + therapist)
1	30	30	€25,119
2	30	15	€15,733
3	30	10	€12,604
4	30	7,5	€11,039

**Table 4:** Additional Protocol costs. The additional weekly hours are calculated considering two 25-minute daily sessions per 5 days a week (50 min × 2 = 250 min = 4,17 hours).

Additional Protocol costs				
Week (number)	Number of robots per therapist	Hourly cost (robot + therapist)	Additional weekly hours	Additional weekly cost
1	1	€25,119	4,17	€104,74
2	2	€15,733	4,17	€65,61
3	3	€12,604	4,17	€52,56
4	3	€12,604	4,17	€52,56
5	3	€12,604	4,17	€52,56
			Total	**€328,04**

**Table 5:** Mixed Protocol—phase one. Costs of the additional robotic training sessions delivered in the first two weeks.

Mixed Protocol—phase one	
Additional weeks (no.)	2
Days per week (no.)	5
Sessions per day (no.)	2
Session's length (min)	25
Additional sessions (no.)	20
Additional hours (no.)	8,33
Supervised robot (no.)	1-2
Hourly cost (average)	€20,426
*Additional cost *	*€170,21 *

**Table 6:** Mixed Protocol—phase two. The number of substitution hours (24,49) has been calculated by dividing the cost of the first two weeks of additional therapy (€170,21) by the hourly saving (€6,95) determined by the substitutive therapy. The so-calculated substitution hours correspond to 58,78 sessions lasting 25 minutes each, which can be delivered (twice a day) in less than 6 weeks to pay back the cost of the additional therapy.

Mixed Protocol—phase two	
Supervised robot (no.)	3-4
Hourly cost (average)	€11,822
Hourly physiotherapist cost	€18,773
*Hourly saving *	*€6,95 *
Substitution hours (no.)	24,49
Substitution sessions (no.)	58,78
Substitution weeks	5,78
